# Anti-Inflammatory Activity of the Wild Mushroom, *Echinodontium tinctorium,* in RAW264.7 Macrophage Cells and Mouse Microcirculation

**DOI:** 10.3390/molecules24193509

**Published:** 2019-09-27

**Authors:** Sumreen Javed, Wai Ming Li, Mehreen Zeb, Almas Yaqoob, Linda E. Tackaberry, Hugues B. Massicotte, Keith N. Egger, Peter C.K. Cheung, Geoffrey W. Payne, Chow H. Lee

**Affiliations:** 1 Chemistry and Biochemistry Program, University of Northern British Columbia, Prince George, BC V2N 4Z9, Canada; sumreen.javed@alumni.unbc.ca (S.J.); mlidoudou@yahoo.ca (W.M.L.); zeb@unbc.ca (M.Z.); yaqoob@unbc.ca (A.Y.); 2 Ecosystem Science and Management Program, University of Northern British Columbia, Prince George, BC V2N 4Z9, Canada; tackaberry.linda@gmail.com (L.E.T.); hugues.massicotte@unbc.ca (H.B.M.); keith.egger@unbc.ca (K.N.E.); 3 Food and Nutritional Sciences Program, School of Life Sciences, The Chinese University of Hong Kong, Shatin, New Territories, Hong Kong, China; petercheung@cuhk.edu.hk; 4 Northern Medical Program, University of Northern British Columbia, Prince George, BC V2N 4Z9, Canada; Geoff.Payne@unbc.ca

**Keywords:** *Echinodontium tinctorium*, polysaccharide, anti-inflammation, mushroom, British Columbia

## Abstract

The aim of this study was to investigate the anti-inflammatory activity of a previously un-studied wild mushroom, *Echinodontium tinctorium*, collected from the forests of north-central British Columbia. The lipopolysaccharide (LPS)-induced RAW264.7 macrophage model was used to study the in vitro anti-inflammatory activity. The crude alkaline extract demonstrated potent anti-inflammatory activity, and was further purified using a “bio-activity-guided-purification” approach. The size-exclusion and ion-exchange chromatography yielded a water-soluble anti-inflammatory polysaccharide (AIPetinc). AIPetinc has an average molecular weight of 5 kDa, and is a heteroglucan composed of mainly glucose (88.6%) with a small amount of galactose (4.0%), mannose (4.4%), fucose (0.7%), and xylose (2.3%). In in vivo settings, AIPetinc restored the histamine-induced inflammatory event in mouse gluteus maximus muscle, thus confirming its anti-inflammatory activity in an animal model. This study constitutes the first report on the bioactivity of *Echinodontium tinctorium*, and highlights the potential medicinal benefits of fungi from the wild forests of northern British Columbia. Furthermore, it also reiterates the need to explore natural resources for alternative treatment to modern world diseases.

## 1. Introduction

Fungi represent a major and largely untapped source of potentially powerful new pharmaceutical natural products. Despite this, there have been relatively few studies directed at finding medicinal compounds from forest fungi in British Columbia (BC), Canada. To this end, we have begun to explore medicinal properties of BC native fungi, focusing first on three bioassays that are relevant to cancer: growth-inhibitory, immuno-stimulatory, and anti-inflammatory activities [1,2].

The fungus selected for the present study was *Echinodontium tinctorium* (Ellis & Everh.) Ellis & Everh. It is also known as Indian paint fungus due to its distinctive red brick color, and its primary use by Indigenous peoples was in preparing red paint pigments [3]. *Echinodontium tinctorium* is a white rot, able to decay both wood lignin and cellulose. It produces a woody, large hydnaceous basidiocarp or conk [4,5] that typically grows beneath dead branch stubs on live tree trunks [6]. Most studies pertaining to *E. tinctorium* relate to its taxonomy, disease cycle, and management [4,5,7,8,9], but this conk also has a history of use as an anti-bacterial agent by Indigenous peoples [10].

A recent comprehensive study on the DNA sequences spanning ITS1-5.8S-ITS2 and the D1-D2 domains of 28S rDNA showed that the genus *Echinodontium* is represented by *E. tinctorium*, *E. ryvardenii*, and *E. tsugicola*, while a new genus, *Echinodontiellum*, was proposed to accommodate *E. japonicum* [8]. To date, there has only been one compound, echinodol, a triterpene acetate, isolated from *E. tinctorium* [11]. However, the bioactivity of this triterpene acetate was not studied, nor has there been any other study on extracts isolated from this fungus. On the other hand, a number of sesquiterpenes have been isolated from *E. tsugicola*, but only two show weak anti-bacterial activity [12,13,14]. Two sesquiterpenoids were also isolated from *E. japonicum*, but they proved to have no anti-bacterial activity [15].

Inflammation is a component of the body’s own immune response that combats the invasive pathogens, irritants, and cell debris. At the system level, inflammation causes fever, swelling, and pain. At the cellular level, it causes vasodilation and compromises vascular permeability. While the initial intention of an inflammatory event is to support the human body, a prolonged and excessive inflammation is correlated with various clinical presentations like arthritis, cardiovascular complications, and tumor progression [16]. Non-steroidal anti-inflammatory drugs (NSAIDS) are used as first line therapy against inflammation, but their long-term use is associated with gastrointestinal bleeding and other complications, including peptic ulcers [16,17]. This suggests a need to explore alternative medicines, especially natural products that could provide potentially safer options to treat inflammation [16].

The aim of this study was to explore the medicinal bioactivity of *E. tinctorium* collected from north-central BC. Dried and pulverized mushroom fruiting bodies were sequentially extracted using 80% ethanol, 50% methanol, water, and 5% NaOH, a modified approach used to extract anti-tumor polysaccharide and described previously [18]. The extracts were studied for immuno-stimulatory and anti-inflammatory activities, based on their ability to either stimulate TNF-α production or inhibit lipopolysaccharide-induced TNF-α production, respectively, in RAW264.7 mouse macrophage cells. The potent extract for anti-inflammatory activity was purified using a two-step “bioactivity-guided-purification” approach with the help of size-exclusion and ion-exchange chromatography. Gas chromatography (GC) with a flame ionization detector (FID) was employed to analyze the monosaccharide composition. Intravital microscopy coupled with conducted vasodilation measurements in mice gluteus maximus muscle (second order arterioles) were used to confirm its anti-inflammatory activity in vivo. Thus, the main purpose of this study was to investigate the anti-inflammatory activity of *E. tinctorium* in both RAW264.7 cells and mice microcirculation.

## 2. Results and Discussion

### 2.1. Identification of Echinodontium tinctorium

Based on morphological characteristics, the collected fungi were tentatively identified as *Echinodontium tinctorium*. DNA sequencing analysis of the ITS2 region and the 5′ end of the D1-D2 region of the 28S gene using the consensus sequence of the ITS3 and NLB4 primers, followed by a BLAST search of GenBank, further confirmed this finding. The DNA sequence of collections CL37 and CL103 were identical, 481 bp in length, and had highest sequence similarity to collections of *E. tinctorium* from the USA (GenBank KF996509.1: 100% identity/62% coverage) and Canada (GenBank AY854088.1: 99% identity/66% coverage); neither GenBank entry extended into the 28S D1/D2 region, hence the lower percent coverage.

### 2.2. Chemical Extraction and Assessment of Crude Extracts of E. tinctorium for Bio-Activities

**Figure 1:**
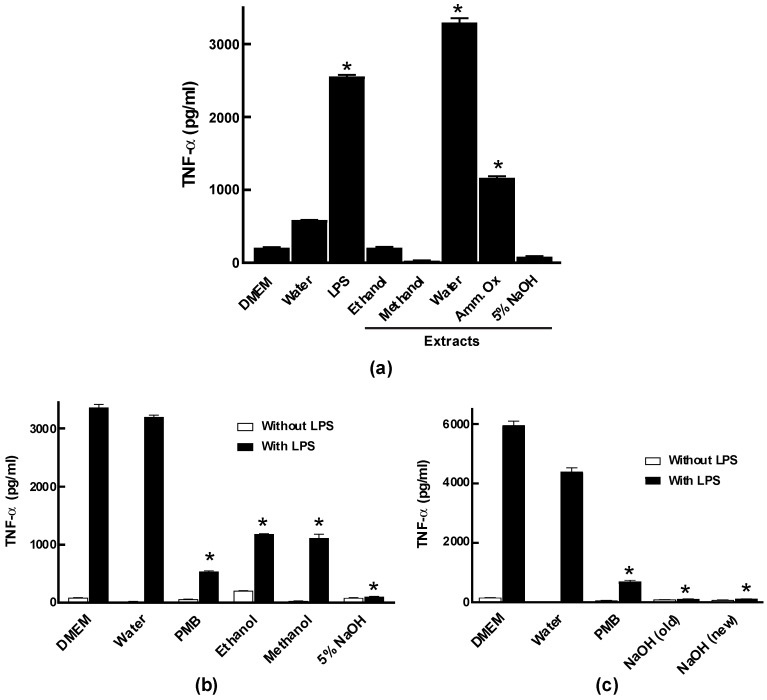
Immuno-modulatory activities of crude extracts obtained from *Echinodontium tinctorium.* (**a**) At 1 mg/mL, crude extracts (80% ethanol, 50% methanol, water, 2% ammonium oxalate, and 5% NaOH) were assessed for their ability to induce TNF-α production in RAW264.7 macrophage cells as an indicator of immuno-stimulation. Lipopolysaccharide (LPS) was used as positive control, whereas media and water were used as negative controls. (**b**) Crude extracts (1 mg/mL) that were inactive for immuno-stimulation (80% ethanol, 50% methanol, and 5% NaOH) were tested for their ability to inhibit LPS-induced TNF-α production as an indicator of anti-inflammatory activity. Polymyxin B (PMB; an inhibitor of LPS) was used as a positive control, whereas media and water treated with LPS were used as negative controls. (**c**) Comparison of the anti-inflammatory activity of 5% NaOH extract from two different collections of *E. tinctorium.* Due to the large quantity of a new collection, the new NaOH extract was used for further purification studies and in vivo experiments. Data shown were pooled from three biological replicates (*n* = 3). Error bars are S.D., and one-way ANOVA was performed as statistical analysis. * denotes *p* < 0.05 as compared to the water control using Sidak’s multiple comparison test.

Powdered *E. tinctorium* was sequentially extracted with 80% ethanol, 50% methanol, water, 2% ammonium oxalate and 5% NaOH, and assessed for immuno-stimulatory activity as shown in Figure 1a. At 1 mg/mL, the water extracts showed potent activity that was stronger than the positive control lipopolysaccharide (LPS), while the ammonium oxalate extract showed modest immuno-stimulatory activity. The 80% ethanol, 50% methanol, and 5% NaOH extracts showed little to no immuno-stimulatory effects and were further assessed for anti-inflammatory activity in vitro (Figure 1b). Both the 80% ethanol and 50% methanol extracts showed potent activity in inhibiting LPS-induced TNF-α production, however, the 5% NaOH extract was the strongest among the three extracts. We subsequently omitted the ammonium oxalate extraction step with the new collection of *E. tinctorium* and proceeded with 5% NaOH extraction after water extraction [1,2]. As shown in Figure 1c, the new 5% NaOH extraction method (without ammonium oxalate extraction) has potent anti-inflammatory activity that is similar to the old 5% NaOH extract.

### 2.3. Purification of Anti-Inflammatory Polysaccharide from E. tinctorium

**Figure 2:**
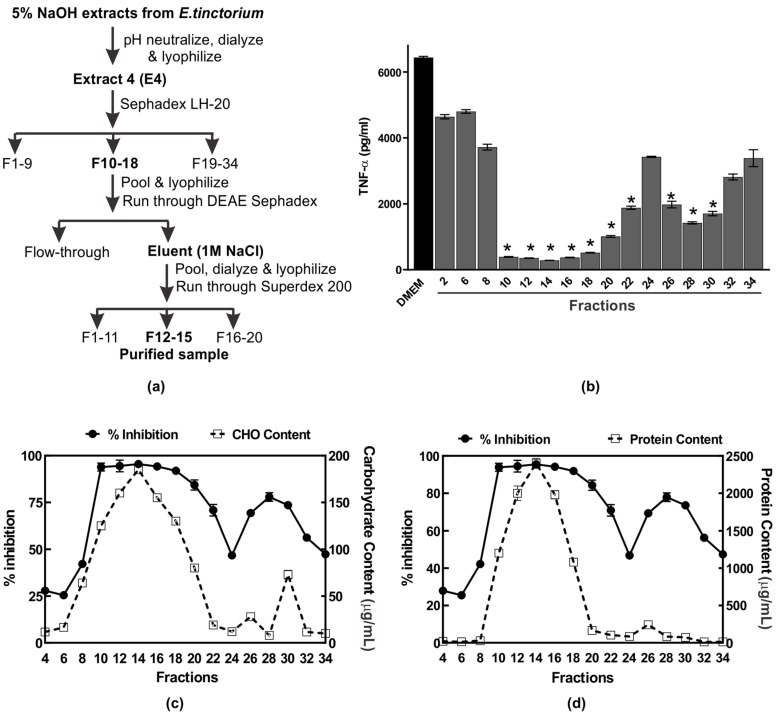
Purification of the anti-inflammatory polysaccharide from *Echinodontium tinctorium* (AIPetinc). (**a**) Summary of the purification scheme used; (**b**) Profile of anti-inflammatory activity of the NaOH extract after elution from Sephadex™ LH-20 column. The assays were performed in triplicate and the data shown is a representative from three biological replicates (*n* = 3). Error bars represent standard deviation. One-way ANOVA was performed as described in Materials and Methods. * denotes *p* < 0.05 as compared to DMEM using Sidak’s multiple comparison test. Fractions which contained anti-inflammatory activity (expressed as % inhibition) were also assessed for carbohydrate (**c**) and protein (**d**) contents. The data shown in (**c**) and (**d**) also result from three biological replicates (*n* = 3).

The 5% NaOH extract was selected for further purification and characterization of the anti-inflammatory compound. A summary of the scheme used to purify the anti-inflammatory compound AIPetinc is shown in Figure 2a. We first subjected the NaOH extract to Sephadex LH-20 chromatography and, as shown in Figure 2b, fractions 10–22 and 26–30 possessed potent anti-inflammatory activity. The first anti-inflammatory activity peak (fractions 10–22) correlated well with the maximal carbohydrate (Figure 2c) and protein (Figure 2d) content, suggesting that the bioactive compound may contain polysaccharide as well as protein. The second anti-inflammatory activity peak (fractions 26–30) correlated well with the maximal carbohydrate (Figure 2c), but not the protein content (Figure 2d), suggesting that the bioactive compound is composed predominantly of polysaccharide.

**Figure 3:**
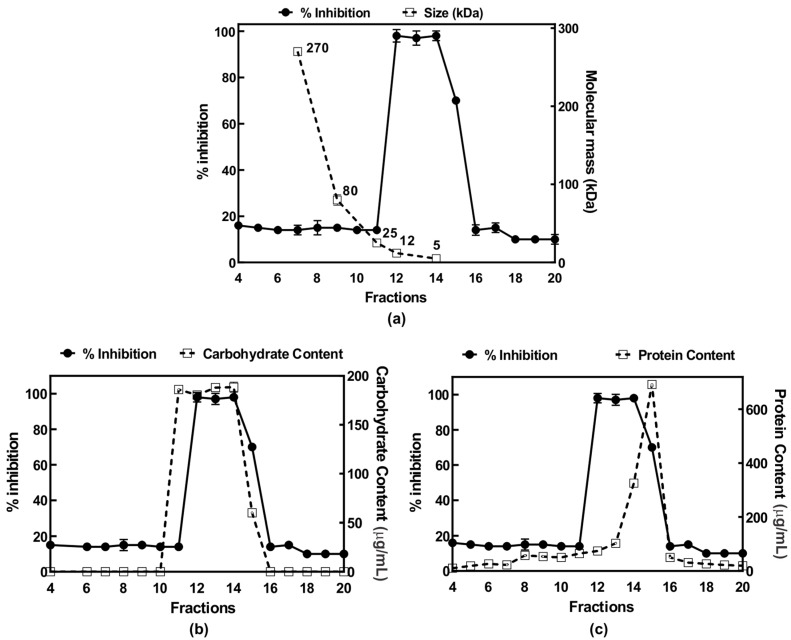
Purification of the anti-inflammatory polysaccharide from *Echinodontium tinctorium* (AIPetinc) using Superdex 200. Fractions collected were assessed for anti-inflammatory activity where percent inhibition represents the inhibition of LPS-induced TNF-α plotted against (**a**) dextran standards, (**b**) carbohydrate contents, and (**c**) protein contents. Results are representative from two separate experiments (*n* = 2).

Several large-scale Sephadex LH-20 runs were performed and fractions which contained the first anti-inflammatory activity peak were pooled, lyophilized, and dose-dependently assessed for activity. When assessed at 0.1–1 μg/μL, the Sephadex LH-20 purified sample had higher anti-inflammatory activity than the crude 5% NaOH E4 extracts (data not shown), demonstrating the efficiency of Sephadex LH-20 in purifying the anti-inflammatory compound. We then assessed several buffers, each with different pH, for possible use with DEAE Sephadex in purifying the anti-inflammatory compound. Amongst the five buffers (*N*-methyl piperazine pH 4.5, Piperazine pH 5.5, l-Histidine pH 6.1, Tris pH 7.7, and diethanolamine pH 8.7) tested, we determined that 20 mM Piperazine pH 5.5 was the most effective buffer for purification of the anti-inflammatory polysaccharide in that the bound bioactive polysaccharide could be eluted with 1 M NaCl (data not shown). Finally, we subjected the bioactive polysaccharide eluted with 1 M NaCl from the DEAE Sephadex column to a higher resolution size-exclusion chromatography, Superdex 200. Results of the analysis of fractions collected from Superdex 200 are shown in Figure 3. The anti-inflammatory activity peak obtained from fractions 12–15 is estimated to have a size of 5 kDa based on the dextran standards (Figure 3a). The activity peak was found to correlate well with carbohydrate content analysis (Figure 3b), but not with protein content analysis (Figure 3c). These results suggest that AIPetinc is a polysaccharide and that the purification steps following Sephadex LH-20 were able to further purify the anti-inflammatory compound.

### 2.4. AIPetinc Inhibited LPS-Induced NO Production and Histamine-Induced TNF-α Production in RAW264.7 Macrophage Cells

**Figure 4:**
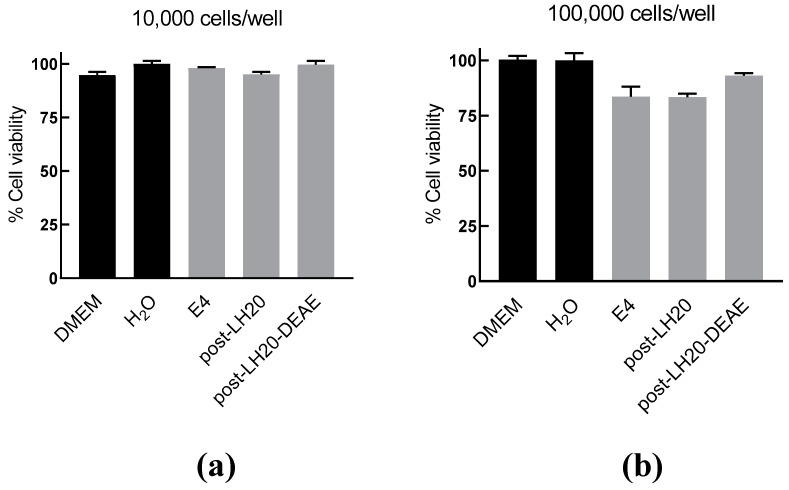
MTT assay demonstrating mild toxicity of AIPetinc on RAW264.7 cells. RAW264.7 cells plated at 10,000 cells/well (**a**) and at 100,000 cells/well (**b**) were incubated with crude (E4) and semi-purified extracts (post-LH20 and post-LH20-DEAE) of *E. tinctorium* for six hours before the MTT assay was performed to determine cell viability. Error bars are S.D., obtained from two biological replicates (*n* = 2).

To understand the mechanism of the anti-inflammatory activity AIPetinc, we first performed MTT assays to test for potential toxicity of the polysaccharide on RAW264.7 cells. As shown in Figure 4a, at a cell density of 10,000 cells/well and incubated for six hours, the normal incubation period to assess anti-inflammatory response, cell viability was similar amongst all groups (Figure 4a). At a cell density normally used in the anti-inflammatory assay (100,000 cells/well), there was a slight decrease in cell viability for the crude extract (E4), and the semi-purified fractions post LH-20 and post-LH20-DEAE that contained AIPetinc (Figure 4b). The small reduction in cell viability (7–16%) obtained for these fractions cannot account for the substantial inhibition of inflammatory response (>90%) observed in Figure 3. As such, the inhibition of LPS-stimulated TNF-α response from RAW264.7 cells cannot be attributed to the mild cytotoxic effects of AIPetinc.

**Figure 5:**
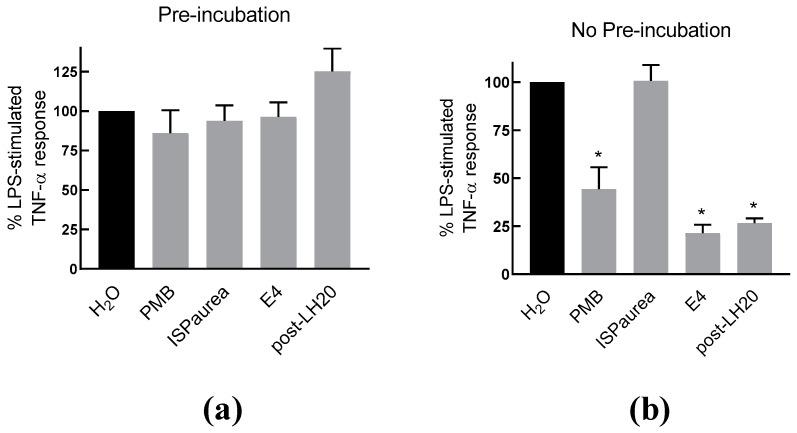
The inhibitory effect of AIPetinc on LPS-stimulated TNF-α response requires co-incubation with LPS. (**a**) LPS-stimulated TNF-α production in RAW264.7 cells with pre-incubation of mushroom extracts isolated from *P. aurea* (ISPaurea) or *E. tinctorium* crude extract (E4) and the semi-purified fraction post-LH20. After incubation for six hours with the indicated extracts and controls, the cells were washed two times before LPS was added for a further six hours incubation. TNF-α production in the medium was then measured. (**b**) LPS-stimulated TNF-α production in RAW264.7 cells co-incubated with mushroom extracts. Error bars are S.D., obtained from three biological replicates (*n* = 3), and one-way ANOVA was performed as statistical analysis. * denotes *p* < 0.05 as compared to H_2_O control using Sidak’s multiple comparison test.

To further examine the anti-inflammatory response of AIPetinc in RAW264.7 cells, we performed an experiment, where RAW264.7 cells were pre-incubated with the extracts before exposure to LPS. The rationale was to test whether AIPetinc is similar to polymyxin B (PMB) that binds to LPS. As shown in Figure 5a, the TNF-α response indeed was not changed when the cells were pre-incubated with PMB, washed, and then stimulated with LPS. However, when PMB was co-incubated with LPS, a substantial decrease in TNF-α response was noted (Figure 5b). This is because PMB is required to bind to LPS extracellularly to prevent LPS binding to the Toll-Like Receptor 4. Similarly, the crude extract (E4) and semi-purified fraction (post-LH20) containing AIPetinc also had no effect on the TNF-α response to LPS, when they were pre-incubated with the RAW264.7 cells (Figure 5a), suggesting that the action of AIPetinc also acts like PMB extracellularly to inhibit LPS binding to its receptor. Since it can be viewed that all polysaccharides have the same potential to neutralize LPS extracellularly, we included another polysaccharide from a different mushroom species, *Phaeolepiota aurea*, for comparison. As shown in Figure 5b, ISPaurea, which is a polysaccharide with glucose as the major component similar to AIPetinc (see below), does not inhibit LPS-stimulated TNF-α response. Thus, although many polysaccharides have been reported to have anti-inflammatory activity, their action cannot be simply attributed to the general structure of the polysaccharide as a β-glucan.

**Figure 6:**
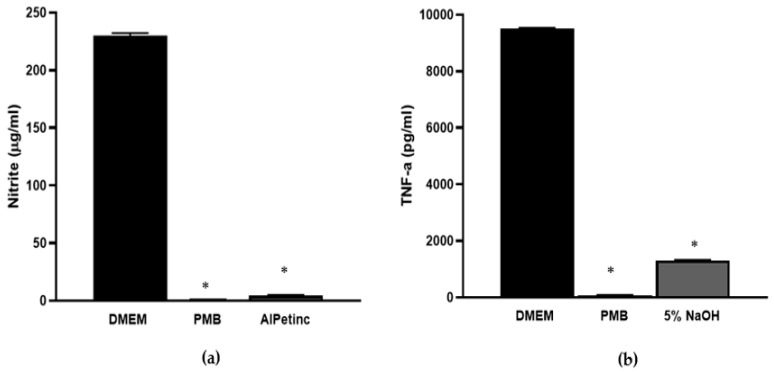
In vitro anti-inflammatory activity of AIPetinc. (**a**) At 1 mg/mL, AIPetinc inhibited LPS-induced nitric acid production in RAW264.7 macrophage cells. PMB was used as a positive control for anti-inflammatory response. Purified sample inhibited ≥ 98% of stimulated nitric oxide (NO). (**b**) At 1 mg/mL, 5% NaOH extract containing AIPetinc inhibited histamine-induced TNF-α production in RAW264.7 macrophage cells. The histamine concentration used is 10^−6^ M. Error bars are S.D., data obtained from three biological replicates (*n* = 3), and one-way ANOVA was performed as statistical analysis. * denotes *p* < 0.05 as compared to DMEM using Sidak’s multiple comparison test.

To further assess the anti-inflammatory potential of AIPetinc, we determined its effect on nitric oxide (NO) production. NO, a critical biomarker for inflammation, plays a major role in chronic inflammation and carcinogenesis [16,19]. It is produced by inducible nitric oxide synthase upon induction by the transcription factor NF-κβ that is a downstream target of LPS and TNF-α. As shown in Figure 6a, 1 mg/mL of AIPetinc significantly inhibited NO production in response to LPS, further confirming the anti-inflammatory activity of AIPetinc.

Histamine, a commonly used inflammation inducer, was dose-dependently assessed for its ability to induce TNF-α production in RAW264.7 cells. At 1 µM, histamine was found to strongly induce TNF-α production (data not shown). Figure 6b shows that indeed, at 1 mg/mL, the 5% NaOH AIPetinc extract could significantly inhibit histamine-induced TNF-α production. The in vitro results using RAW264.7 cells suggest that AIPetinc behaves similarly to PMB, an antibiotic that binds to LPS to neutralize it from stimulating an innate immune response. It remains to be tested whether AIPetinc has bactericidal effects by binding to LPS on bacterial cell walls.

### 2.5. AIPetinc Blocked Histamine-Induced Inflammatory Event in Mice Microcirculation

**Figure 7:**
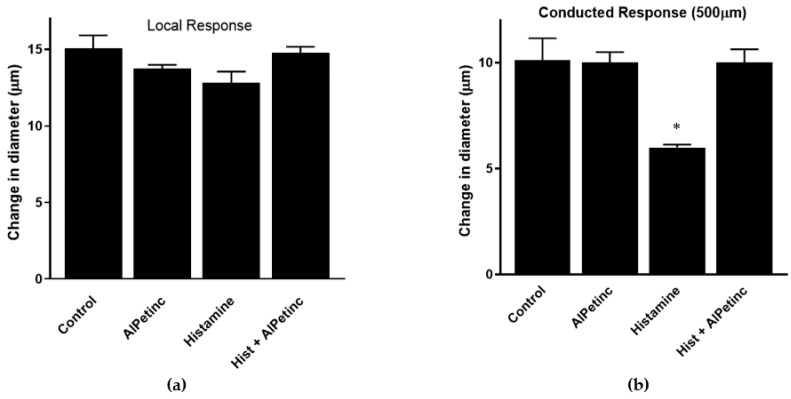
In vivo anti-inflammatory activity of AIPetinc. (**a**) Local response to conducted vasodilation in intact arterioles of C57BL6 mice (*n* = 10). Responses to acetylcholine (Ach) (1psi, 500 ms) were recorded at the site of stimulation (local: distance = 0). There was no significant difference in the local response in arterioles of C57BL6 mice under different treatment. (**b**) Responses to ACh (1psi, 500 ms) were recorded upstream (with respect to blood flow) at a distance of 500 µm (conducted response). At each site, change in diameter was calculated as peak response diameter minus resting diameter. There is a significant difference between conducted response of histamine vs. histamine + AIPetinc indicating the anti-inflammatory potential of the molecule. Error bars represents mean ± S.D. One-way ANOVA was performed as described in methods. * denotes *p* < 0.05 as compared to control.

Inflammation is a multi-step physiological response that begins with the stimulation of the immune system to release cytokines leading to localized changes in the vasculature, including vasodilation and increased permeability of the blood vessel [20,21,22]. Results obtained from RAW264.7 cells suggest that AIPetinc is capable of inhibiting the release of cytokine from macrophages, an early event of an inflammatory response. To further investigate the anti-inflammatory potential of AIPetinc, we used an in vivo intravital microscopy mouse model system to study acetylcholine (ACh)-induced conducted vasodilation in the microcirculation as a measure of vasculature integrity, a late event in inflammation. Previous studies have shown that histamine, a strong inflammation mediator in the blood vessel, can inhibit ACh-induced vasodilation at a distance from the stimulation site, attributed to histamine’s inhibitory effect on cell-to-cell coupling through a mechanism which is dependent on NO production [22]. Figure 7a shows that the local diameter change at the site of delivery was almost identical in the second order (2A) arterioles under the different treatment groups, whereas Figure 7b demonstrates that histamine decreased the vasomotor response to ACh at 500 µm upstream from the site of ACh treatment. This lack of change at the “local” site indicates the arterioles are intact and viable, implying a decrease in cellular communication (loss of vascular integrity via inflammation) along the arterioles. The change in diameter dropped from 10 ± 0.3 µm in the control state to 6 ± 0.06 µm in the inflammatory state induced by histamine. The addition of AIPetinc reversed the response to histamine, suggesting cellular communication was restored by AIPetinc. Results strongly suggest that AIPetinc has anti-inflammatory activity in the microcirculation to restore vasculature integrity, possibly due to the ability of AIPetinc to reduce NO production. This, however, needs to be confirmed by further experimentation. The study on AIPetinc and our previous study on extracts from the fungus *Inonotus obliquus* [23] demonstrate that inhibition of LPS-induced TNF-α production in mouse macrophage RAW264.7 cells is a good biological system to study the anti-inflammatory activity in vitro. Furthermore, the mouse microcirculation established here could also be used as a model to study the in vivo action of anti-inflammatory compounds from medicinal fungi in the vasculature.

### 2.6. Chemical Analysis of the Anti-Inflammatory Polysaccharide from E. tinctoirum

The monosaccharide composition of AIPetinc was determined by Gas Chromatography (GC). For comparison, we also performed parallel experiments on the well-known polysaccharide-protein complex isolated from *Trametes versicolor*, polysaccharide-K [24]. Table 1 shows that AIPetinc consisted mainly of glucose (88.6%). Other minor monosaccharides present included galactose (4.0%), mannose (4.4%), fucose (0.7%), and xylose (2.3%). This is different from polysaccharide-K, which has a relatively higher amounts of mannose (14.0%) and galactose (7.1%), and from a growth-inhibitory polysaccharide recently isolated from *Paxillus involutus*, which has a relatively high amount of galactose (20.8%) (Table 1) [25]. A complementary approach, the β–glucan assay kit, was also used to assess the amount of α– and β–glucans present in AIPetinc. Results, summarized from two separate experiments, show that the total glucan in AIPetinc has 93.42% β–glucan and 6.57% α–glucan. Overall, our results suggest that AIPetinc belongs to the family of β–glucans with anti-inflammatory activity [26,27].

## 3. Materials and Methods

### 3.1. Materials and Chemicals

*Echinodontium tinctorium* was collected from three separate locations in north-central BC: an old growth forest near McBride (voucher not kept); Twin Falls Recreation Site, Smithers (CL37); and Pine Lake Recreation Site, Terrace (CL103). DEAE Sephadex, dextran standards (T1, T5, T12, T25, T50, T80, T150, T270, T410), and the standard monosaccharides (glucose, mannose, rhamnose, ribose, xylose, arabinose, fucose, galactose, galactosamine, and glucosamine) were purchased from Sigma-Aldrich (St. Louis, MO, USA). Fetal bovine serum was from Life Technologies Inc. (Waltham, MA, USA), and Dulbecco Modified Eagle Medium was from LONZA (Walkersville, MD, USA). Sephadex LH-20 resin and pre-packed Superdex 200 Increase 10/300 GL were from GE Healthcare (Uppsala, Sweden). Polysaccharide-K was purchased from Kureha Pharmaceuticals (Tokyo, Japan). All other reagents used were of analytical grade.

### 3.2. Identification of Mushroom Species

The mushroom collections were initially identified as *Echinodontium tinctorium* based on morphological characteristics [3,6]. Two (CL37 and CL103) of the three collections were confirmed by DNA sequencing. Small tissue samples were aseptically collected from fungal sporocarps and dried. DNA was extracted using a PowerSoil DNA isolation kit according to the manufacturer’s protocol. Extracted DNA samples underwent polymerase chain reaction (PCR) amplification as described previously [1,2] using primers ITS3 (5′-GCATCGATGAAGAACGCA-3′) and NLB4 (5′-GGATTCTCACCCTCTATGA-3′). DNA sequences of the Internal Transcribed Spacer 2 (ITS2) and the 5′ end of the D1-D2 region of the 28S gene were aligned and edited using CLC Main Workbench (Qiagen, Carlsbad, CA, USA), then entered into the Basic Local Alignment Search Tool (BLAST) to determine closest sequence identity in GenBank.

### 3.3. Extraction and Purification of Anti-Inflammatory Polysaccharide

The fungal specimens were dried and then crushed into powder form using a Hammer mill. The material was sequentially fractionated into four extracts as previously described [1,2]; this is an extension of a well-established protocol for extraction of anti-tumor polysaccharides [18]. Typically, about 1 g of powdered specimen was extracted with 10 mL of 80% ethanol for 3 h at 65 °C. The solution, filtered through Whatman paper No.3, was then referred to as Extract 1, while the residue was subjected to the second step: 50% methanol extraction for 3 h at 65 °C. The filtrate (Extract 2) was kept, while the residue was subjected to the third step: water extraction for 6 h at 65 °C. The filtrate from the water extraction step was referred to as Extract 3. Finally, this residue was subjected to the last step: 5% sodium hydroxide (NaOH) extraction at 65 °C for 6 h. The filtered solution from this step was referred to as Extract 4. Initially, 2% ammonium oxalate extraction was also performed at the fourth step, but it was later omitted because of the incompatibility of oxalate with the medium used for growing RAW264.7 cell lines. In the initial trials, extract 4 represents 2% ammonium oxalate, and extract 5 represents the 5% NaOH fraction. A subsequent collection of *Echinodontium tinctorium* was extracted without the ammonium oxalate step. All extracts were subjected to roto-evaporation followed by lyophilization. All lyophilized extracts were reconstituted in water at 2 mg/mL and filter-sterilized using a 0.2 μm filter (Sarstedt, QC, Canada) before they were assessed for immuno-stimulatory or anti-inflammatory activities, as described below. Some lyophilized extracts were reconstituted in methanol to solubilize the extract as indicated.

All extracts were tested for their ability to stimulate TNF-α in RAW264.7 cells. Extracts negative for the immuno-stimulatory activity were then assessed for their ability to inhibit LPS-induced TNF- α as a measure of their anti-inflammatory potential, as described previously [1,2]. The crude alkaline extract (NaOH extract) with anti-inflammatory activity was suspended in water and centrifuged at 100× *g* for 5 min to remove any insoluble particulate. Two percent (or 1.12 mL supernatant) was loaded onto a size exclusion, Sephadex LH-20 column (16 mm × 70 cm), and run at a flow rate of 1.0 mL/min. Thirty-five 2 mL fractions were collected and assessed for anti-inflammatory activity by ELISA, as described in Section 3.6. Carbohydrate and protein contents in the fractions were determined using the phenol-sulfuric acid method [28] and Pierce BCA protein assay kit (Waltham, MA, USA), respectively. Fractions with potent activity were pooled, lyophilized, resuspended in water and loaded onto DEAE Sephadex in XK-50 column (50 mm × 100 cm) equilibrated with 20 mM piperazine buffer pH 5.5, and ran at a flow rate of 1.0 mL/min. After washing the column with the same buffer, the column was eluted with two bed volumes (2 L) containing 1 M NaCl. The collected eluent was lyophilized, dialyzed, re-lyophilized, and reconstituted in water at a concentration of 2 mg/mL before loading 500 μL onto a Superdex 200 larger column (1.0 × 30 cm), which was run at a flow rate of 1 mL/min. The bioactive eluent collected in fractions from Superdex 200 was pooled, lyophilized, and designated as AIPetinc.

### 3.4. Monosaccharide Composition Analysis

The monosaccharide composition of AIPetinc was determined by GC analysis. Briefly, samples were subjected to sequential acid hydrolysis (12 M sulfuric acid for 1 h at 35 °C, and then 2 M sulfuric acid for 1 h in a boiling water bath). Alditol acetates of the neutral sugars in the acid hydrolysate were prepared according to the previously described method [29] with β-d-allose as the internal standard. Alditol acetates of the monosaccharides were quantified by an HP6890 series II gas chromatography, using an Alltech DB-225 capillary column (15 m × 0.25 mm i.d., 0.25 μm film, (Analytical Columns, New Addington, Croydon, England) with the following oven temperature program: initial temperature, 170 °C; temperature rise at 2 °C/min to 220 °C and final hold for 15 min. The carrier gas was helium, and detection was made by flame ionization.

### 3.5. Determining the Percentage of α- and β-Glucans in AIPetinc

The β–glucan content of AIPetinc was determined using β–Glucan Assay Kit (Megazyme, Ireland) with slight modification. Ten milligrams of AIPetinc and the yeast positive control were first subjected to total glucan determination as according to the manufacturer’s instructions. Two hundred microliters of ice cold 12 M sulfuric acid were added and the mixtures were incubated for 2 h in an ice-water bath with occasional mixing. Four hundred microliters of MilliQ water (Millipore Canada, Etobicoke, ON, Canada) were added and mixed for 10 s, followed by the addition of 600 μL of MilliQ water and mixing and incubation at 100 °C for 2 h. After cooling to room temperature, 100 μL of mixtures were transferred to a 10 mL volumetric flask, and the remaining mixtures in the vial were rinsed with 5 mL 200 mM sodium acetate buffer (pH 5) prior to transferring to the 10 mL volumetric flask. Six hundred microliters of 10 M KOH solution were pipetted to the volumetric flask, and the volume was adjusted with 200 mM sodium acetate buffer (pH 5). The contents were mixed and transferred to a 15 mL tube and centrifuged at 1500× *g* for 10 min. A total of 10 μL of the supernatant (in duplicates) of the centrifuged extract were transferred to a 1.5 mL tube, followed by the addition of 10 μL of a mixture of exo-1,3-β-glucanase (20 U/mL) plus β-glucosidase (4 U/mL) in 200 mM sodium acetate buffer (pH 5.0). The same procedure was performed with a positive control (glucose) and negative control (sodium acetate buffer), with a total of 10 μL each of the 1 mg/mL Glucose Standard solution or 200 mM sodium acetate buffer (pH 5). The resulting solution was then gently vortexed and incubated at 40 °C for 60 min. After one hour, 300 μL of GOPOD reagent was transferred into each tube, followed by incubation at 40 °C for 20 min. Upon completion, 100 μL of the solution was transferred into a 96-well UV plate and the absorbance of all solutions (AIPetinc, yeast control, negative control, and glucose control) was measured at 510 nm against the reagent blank using Synergy 2 multi-plate reader (Bio-Tek, Winooski, VT, USA).

For determination of the amount of α–glucan in samples, approximately 10 mg of AIPetinc and the yeast positive control were mixed with 200 μL of 2 M KOH solution. The vials were then capped and hand-shaken on an ice-water bath for 20 min, followed by the addition of 800 μL of 1.2 M sodium acetate buffer (pH 3.8) and 20 μL of amyloglucosidase (1.630 U/mL) plus invertase (500 U/mL). The vials were then incubated at 40 °C for 30 min with occasional mixing. Subsequently, the contents were transferred into a 1.5 mL tube and centrifuged at 1500× *g* for 10 min. A total of 10 μL of the supernatant (in duplicates) was transferred to a separate 1.5 mL tube, followed with the addition of 10 μL of sodium acetate buffer (200 mM, pH 5.0) plus 300 μL of GOPOD reagent and incubated at 40 °C for 20 min. The same procedure was performed with a positive control (glucose) and negative control (sodium acetate buffer), with a total of 10 μL each of the 1 mg/mL Glucose Standard solution or 200 mM sodium acetate buffer (pH 5), respectively, mixed with 300 μL of GOPOD reagent. Upon completion, 100 μL of the solution was transferred into a 96-well UV plate and the absorbance of all solutions (AIPetinc, yeast control, negative control, and glucose control) was measured at 510 nm against the reagent blank. The total glucan and α–glucan in % *w/w* were calculated according to the manufacturer’s guidelines. The amount of β-glucan was determined by the following simple equation: β-glucan (% *w/w*) = Total glucan (% *w/w*) − α–glucan (% *w/w*).

### 3.6. Cell Line and Assessment for Immuno-Stimulatory Activity

RAW264.7 mouse macrophage cell line was purchased from the American Type Culture Collection (Rockville, MD, USA) and maintained in Dulbecco Modified Eagle Medium. Cells were grown in media supplemented with 10% fetal bovine serum in a humidified incubator at 37 °C supplied with 5% carbon dioxide. To assess immuno-stimulatory activity, we measured TNF-α production in RAW264.7 cells upon treatment with 1 mg/mL fungal extracts for 6 h. Fifty µL of medium was removed and stored at −80 °C until TNF-α content determination using enzyme-linked immunosorbent assay (ELISA), as previously described [1,2].

### 3.7. Anti-Inflammatory Activity In Vitro

To assess anti-inflammatory activity, RAW264.7 cells were induced with 250 ng/mL of lipopolysaccharide (LPS) in the presence or absence of 1 mg/mL of fungal extracts or purified samples for 6 h. Polymyxin B (PMB) (100 units, Sigma) was used as a positive control based on its ability to inhibit LPS [1,2,30,31]. The measurement of TNF-α production was determined using ELISA, as described in Section 3.6. Statistical analysis was performed using one-way ANOVA. Sidak’s or Dunnett’s multiple comparisons test was done as post-hoc analysis once the null hypothesis was rejected.

### 3.8. Nitric Oxide Determination Assay

As nitric oxide (NO) is a short-lived chemical in in vitro settings, for the estimation of inhibition of NO, nitrite levels were measured as representative of NO. The assay absolutely requires the use of RAW264.7 macrophage cells that have not been passaged (divided) extensively. Cells were plated at a density of 100,000 cells/well as described previously. After an overnight incubation, cells were treated with: DMEM alone, 1 µg of LPS, 1 µg of LPS + PMB (100 units), 1 µg of LPS + AIPetinc (1 mg). After 24 h incubation, supernatants were collected and assessed for NO content using Promega^TM^ Griess Reagent System (Promega Corporation, Madison, WI, USA). The protocol was followed as indicated by the supplier with a slight modification. Briefly, NO standards were prepared via serial dilution from stock (0.1 M Sodium Nitrite). One hundred µL of samples were added to each well, followed by 50 µL of Sulphanilamide solution and 50 µL of *N*-1-napthylethylenediamine dihydrochloride solution. The plates were read at 540 nm, and the amount of NO was quantified by interpolating the serial dilution of nitrite (NaNO_2_) levels using PRISM software (version 7, GraphPad Software, San Diego, CA, USA).

### 3.9. Intravital Microscopy and Measurement of Conducted Vasodilation

All procedures were approved by the Institutional Animal Care and Use Committee of the University of Northern British Columbia (2016-9 Payne). Male black mice C57BL6 (average weight ~22–30 g and 8–20 weeks of age, *n* = 10) were purchased from Jackson Laboratories, Bar Harbor, ME, USA. Mice were housed at ~ 24 °C on a 12:12-h light dark cycle with free access to food and water. The surgery and gluteus muscle preparation of black C57BL6 mice have been previously described [23]. Briefly, microcirculation in a mouse gluteus maximus muscle was exposed with precise technique. Vessel was equilibrated with physiological saline solution (PSS) to simulate in vivo conditions and vascular integrity was tested prior to the experiment to avoid any experiment-induced inflammatory event. One second order arteriole (2A) with preserved vascular tone was selected as a test vessel. Resting internal diameter was measured under control conditions (PSS). To evaluate conducted vasodilation response under PSS, the vasomotor response to exogenous Acetylcholine (ACh) was measured at the site of delivery (local) and 500 µm upstream from the site of stimulation (conducted). After measuring the local and conducted response under controlled conditions, the preparation was equilibrated with histamine (10^−5^ M) for 30 min. The internal diameter was measured as the effect of histamine on local and conducted vasodilation. After recording the effect of histamine, the preparation was super-fused separately with AIPetinc (*n* = 5) and the added histamine so that the final concentration was 12.5 µg/mL. We have previously established that at 12.5 μg/mL, the reference methanolic extracts of *Inonotus obliquus* had minimal effects on the vessels [23]. The concentration of the AIPetinc was selected on this same criterion (i.e., the dose at which there was negligible effect on the diameter of 2A arteriole under resting conditions). This mixture (histamine + AIPetinc) was allowed to run on the preparation for 30 min, and the local and conducted response was measured in the same way as described previously while the solution was still running on the muscle preparation. The response to ACh was calculated as the diameter change (i.e., peak response diameters minus the resting diameters). Data was analyzed using One-way ANOVA (PRISM) and is represented as means ± S.D. Value of n refers to the number of arterioles studied. Differences among groups is statistically significant when *p* < 0.05.

## 4. Conclusions

This is the first description of immuno-modulatory activities of wild fungal extracts of *Echinodontium tinctorium* collected in north-central BC, Canada. We showed that the 5% NaOH extract from *E. tinctorium* has potent anti-inflammatory activity. Using Sephadex LH-20, DEAE Sephadex, and Superdex 200, we successfully isolated a 5 kDa anti-inflammatory polysaccharide (AIPetinc). AIPetinc is composed predominantly of β-glucan with small amounts of heteroglycans containing galactose, mannose, fucose, and xylose. The polysaccharide inhibited LPS and histamine-induced TNF-α production, and LPS-induced NO production in RAW264.7 mouse macrophage. It also restored the decreased conducted vasodilation induced by histamine in mouse gluteus maximus muscle, further supporting the anti-inflammatory activity of AIPetinc in vivo. Further mechanistic study is required to identify the mechanism of action and the molecular pathways affected as a result of the anti-inflammatory response.

## Figures and Tables

**Table 1:** Monosaccharide composition of purified anti-inflammatory polysaccharide from *E. tinctorium* (AIPetinc).

Monosaccharide Composition (%)	Anti-Inflammatory Polysaccharide from *E. tinctorium*	Growth-Inhibitory Polysaccharide from *P. involutus* [25]	Polysaccharide-K [24]
Glucose	88.6	65.9	74.4
Galactose	4.0	20.8	7.1
Mannose	4.4	7.8	14.0
Fucose	0.7	3.2	1.6
Xylose	2.3	2.3	2.7
Ribose	-	-	0.2
